# The Role of Nuclear Receptor NHR-64 in Fat Storage Regulation in *Caenorhabditis elegans*

**DOI:** 10.1371/journal.pone.0009869

**Published:** 2010-03-25

**Authors:** Bin Liang, Kim Ferguson, Lisa Kadyk, Jennifer L. Watts

**Affiliations:** 1 School of Molecular Biosciences, Washington State University, Pullman, Washington, United States of America; 2 Exelixis Inc., South San Francisco, California, United States of America; Buck Institute for Age Research, United States of America

## Abstract

Nuclear hormone receptors (NHRs) play vital roles in the regulation of metabolism, reproduction, and development. We found that inactivation of a *C. elegans* HNF4 homologue *nhr-64* by RNA interference (RNAi) suppresses low fat stores in stearoyl-CoA desaturase-deficient *fat-6;fat-7* double mutants and sterol regulatory element binding protein (SREBP) *sbp-1* mutants. Furthermore, inactivation of *nhr-64* improves the growth rate of the *fat-6;fat-7*and *sbp-1* strains. While *nhr-64RNAi* subtly affects fatty acid composition and fat storage in wild-type *C. elegans*, its effects on lipid metabolism are most apparent in the background of stearoyl-CoA desaturase or SREBP deficiency. NHR-64 displays transcriptional activating activity when expressed in yeast, and inactivation of *nhr-64* affects the expression of at least 14 metabolic genes. Wild-type worms treated with *nhr-64* RNAi display increased expression of acetyl-CoA carboxylase as well as increased abundance of *de novo* synthesized monomethyl branched chain fatty acids, suggesting an increase in fat synthesis. However, reduced expression of the acetyl-CoA synthetase gene *acs-2* and an acyl-CoA oxidase gene indicates that a key role of NHR-64 may be to promote fatty acid oxidation in mitochondria and peroxisomes. These studies reveal that NHR-64 is an important regulator of fat storage in *C. elegans*.

## Introduction

Nuclear hormone receptors (NHRs) are transcription factors that respond to lipophilic molecules to regulate the expression of target genes involved in metabolism, reproduction, and development. In mammals, peroxisome proliferator-activated receptors (PPARs), liver X receptors (LXR), hepatocyte nuclear factor 4 (HNF4) and farnesoid X receptor (FXR) are important regulators of lipid metabolism [1]. The genome of nematode *Caenorhabditis elegans* contains 284 NHRs, several of which have been implicated in lipid metabolism [2], [3]. For example, DAF-12, a homologue of the vertebrate vitamin D receptor, responds to its ligand, dafachronic acid, to regulate fat metabolism as well as development, dauer formation, and longevity [4]–[6]. Several NHRs were shown to regulate lipid deposition as indicated by Nile Red staining in a genome-wide screen [7].

The HNF4 class of nuclear receptors is greatly expanded in *C. elegans*, with 269 members. Mutations in human HNF4α are associated with maturity-onset diabetes of the young, an autosomal dominant genetic condition associated with early onset diabetes [8]. *Drosophila melanogaster* encodes only one HNF4 ortholog, and larvae carrying a null mutation in this gene are unable to mobilize fat stores for energy during starvation [9]. Furthermore, the Drosophila mutants display decreased expression levels of genes involved in fatty acid catabolism and oxidation. Two *C. elegans* HNF4α orthologs, NHR-49 and NHR-80, regulate fatty acid desaturation [10], [11]. In addition, NHR-49 regulates fatty acid oxidation and the response of nematodes to fasting [10], [12].

Another key regulator of lipid metabolism is the membrane tethered transcription factor SREBP [13]. It resides in the ER membrane and levels of cellular lipids regulate its cleavage and translocation to the nucleus, where it activates a number of genes involved in lipid synthesis [14], [15]. The mammalian SREBP-1a and SREBP-1c transcription factors stimulate expression of genes involved in fatty acid biosynthesis while SREBP-2 stimulates genes involved in cholesterol biosynthesis. *C. elegans* and *Drosophila melanogaster*, neither of which possess all of the enzymes required for *de novo* cholesterol synthesis, each encode one SREBP isoform required for efficient transcription of genes involved in fatty acid synthesis [16], [17]. Dietary supplementation of monounsaturated fatty acids significantly improves growth of SREBP-deficient larvae in both species [18], [19].

The first step in the production of unsaturated fatty acids is catalyzed by Δ9 desaturase, also known as stearoyl-CoA desaturase (SCD), the enzyme necessary for the insertion of a double bond into a saturated fatty acid. This step is now recognized as a key control point in the regulation of fat homeostasis. Monounsaturated fatty acids are preferred substrates for the biosynthesis of triacylglycerol, phospholipids, and cholesterol esters [20]. In mice, SCD1 deficiency leads to reduced adiposity resulting from increased energy expenditure and decreased lipogenesis, as well as to resistance to diet-induced weight gain [21]. *C. elegans* SCD deficiency produces a similar phenotype. Three genes, *fat-5, fat-6*, and *fat-7*, encode Δ9 desaturases in *C. elegans* [22]. Strains carrying mutations in single Δ9 desaturase genes show only subtle defects in fatty acid composition, growth and fertility because expression the remaining isoforms increases to compensate for the mutated activity. In contrast, the *fat-6;fat-7* double mutant strain displays slow growth, reduced fertility, and reduced fat stores and increased expression of genes involved in β-oxidation [23].

Regulation of metabolic homeostasis is very complex, and anabolic and catabolic pathways are continuously being activated or repressed in response to dietary input and energy needs. Because stearoyl-CoA desaturase-deficient mice and nematodes show an increase in fat oxidation and reduced fat storage, we sought to reverse this phenotype by isolating suppressor mutations in *C. elegans* that restore fat stores in the low-fat *fat-6;fat-7* double mutant strain. Suppressor screens are a powerful way to uncover more information about how a particular gene product functions in the context of other cellular proteins and pathways. We used RNA interference (RNAi) to screen the large family of *C. elegans* nuclear receptors for gene inactivations that suppress the low fat and slow growth of stearoyl-CoA desaturase deficient nematodes. We found that reduction of *nhr-64* by RNAi increases fat stores and improves the growth and brood size of *fat-6;fat-7* double mutants. In addition, inactivation of *nhr-64* also partially suppresses the SREBP mutation *sbp-1* and increases fat stores in wild type animals. The suppression of the slow growth and low fat stores in the *fat-6;fat-7* double mutants correlates with lower levels of stearic acid (18∶0) and decreased expression of several β-oxidation genes, indicating that NHR-64 is an important regulator of lipid homeostasis.

## Results

### Nuclear hormone receptor *nhr-64* suppresses low fat of stearoyl-CoA desaturase (SCD)-deficient *C. elegans*

*C. elegans fat-6;fat-7* double mutants, like SCD1 deficient mice, accumulate less fat than wild type and display developmental defects that are due to defective biosynthesis of unsaturated fatty acids [23]. Previous studies indicated that reduction of *nhr-80* and *nhr-49* would enhance *fat-6;fat-7* growth defects, since these two NHRs are required for the induction of *fat-*5, which encodes a palmitic acid-specific Δ9 desaturase activity that partially compensates for *fat-6* and *fat-7* deficiency [10], [11]. However, we suspected that some NHRs may regulate other lipid metabolism pathways that may compensate for the *fat-6;fat-7* defects and therefore improve the *fat-6;fat-7* growth and fat storage defects. We used feeding RNAi to inactivate over 200 genes encoding nuclear hormone receptors. Suppression of the *fat-6;fat-7* defects was scored by examining lipid deposition using Nile Red staining and monitoring growth rate and brood size of *fat-6;fat-7* mutants.

**Figure 1:**
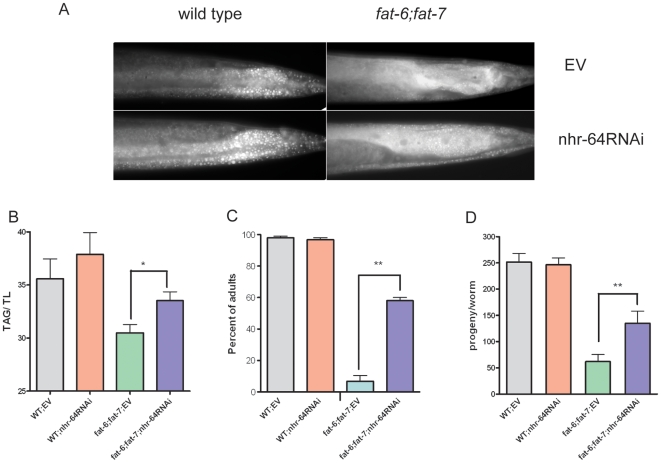
Inactivation of *nhr-64* by RNAi increases fat stores, growth rate, and brood size of *fat-6;fat-7* double mutants grown on *E. coli* strain HT115. Values determined to be significantly different from worms treated with empty vector, *P<0.05, **P<0.01. (A) Inactivation of *nhr-64* increased Nile Red staining of fixed wild type and *fat-6;fat-7* double mutants. Images were captured at using identical settings and exposure time for each image. Animals shown are young adults. Anterior is left, posterior is right. (B) Inactivation of *nhr-64* increased the triacylglycerol/total lipid (TAG/TL) ratio in *fat-6;fat-7* double mutants. Total lipids were extracted from three independent biological replicates and separated into triacylglycerol and phospholipid fractions using thin layer chromatography and quantified using gas chromatography. Error bars are standard deviation. (C) Improved the growth rate of *fat-6;fat-7* double mutants treated with *nhr-64RNAi* and empty vector control (EV). The graph shows the percentage of animals that reached adult stage 72 h after plating synchronized L1 larvae onto RNAi plates seeded with *E. coli* strain HT115 carrying empty vector (EV) or *nhr-64RNAi*. The experiment was repeated twice, each time using 100-150 animals. Error bars show the range of the two experiments. (D) Inactivation of *nhr-64* led to increased brood size in *fat-6;fat-7* double mutants. The number of progeny produced by individual *fat-6;fat-7* and wild-type animals treated with either empty vector (EV) or *nhr-64RNAi* was counted. Data shown are the average brood size of 10–15 individuals. Error bars are standard error.

We found that inactivation of the nuclear hormone receptor gene *nhr-64* led to higher fat stores in *fat-6;fat-7* double mutants as visualized by Nile Red staining of fixed animals (Figure 1A). To verify our observation, we measured the fat content of *nhr-64* worms by separating lipid classes using thin layer chromatography (TLC) and quantifying them using gas chromatography (GC) (Figure 1B). We confirmed our previous findings that *fat-6;fat-7* double mutants have lower fat stores than wild type [23]. Inactivation of *nhr-64* by RNAi increased the TAG content of *fat-6;fat-7* (TAG/total lipid ratio increased from 30.5% to 33.5%). Furthermore, *nhr-64RNAi* showed a trend toward increased fat stores compared to wild type. As expected, we found that *nhr-80* and *nhr-49* caused arrested growth and lethality in the *fat-6;fat-7* strains.

We then quantified the effect of *nhr-64RNAi* on the growth rate and brood size of *fat-6;fat-7* double mutants (Figures 1C and 1D). We counted the percentage of animals at various developmental stages 72 hours after plating synchronized L1s on control bacteria (EV) or on bacteria producing double-stranded RNA corresponding to *nhr-64* (*nhr-64RNAi*). After 72 hours of growth, less than 10% of *fat-6;fat-7* control animals had reached the adult stage. However, greater than 50% of *fat-6;fat-7* animals treated with *nhr-64RNAi* had reached adult stage (Figure 1C). We then transferred L4 animals from both EV and *nhr-64RNAi* plates to corresponding fresh RNAi plates, and scored the number of eggs laid. As reported previously [23], *fat-6;fat-7* double mutants produced a small fraction of the number of progeny produced by wild-type nematodes (Figure 1D). In contrast, inactivation of *nhr-64* significantly increased the brood size of *fat-6;fat-7* animals by more than two fold, from an average of 55 progeny per worm to an average of 127 progeny/worm. Inactivation of *nhr-64* did not cause growth, fertility, and other morphological changes in wild-type nematodes (Figure 1C and 1D). Thus, the lipid metabolism changes brought about by *nhr-64RNAi* are more apparent in the *fat-6;fat-7* double mutants than they are in a wild-type background.

### Inactivation of *nhr-64* suppresses low fat and slow growth of *sbp-1*

Depletion of the *C. elegans* SREBP ortholog, *sbp-1*, results in decreased fat stores, reduced fertility, slow growth, and larval lethality [7], [17], [18], [24]. Since *fat-7* is a target of SBP-1, and dietary oleic acid rescues some of the defects of *sbp-1RNAi* [18], we asked whether inactivation of *nhr-64* also suppresses the *sbp-1*mutation.

**Figure 2 pone-0009869-g002:**
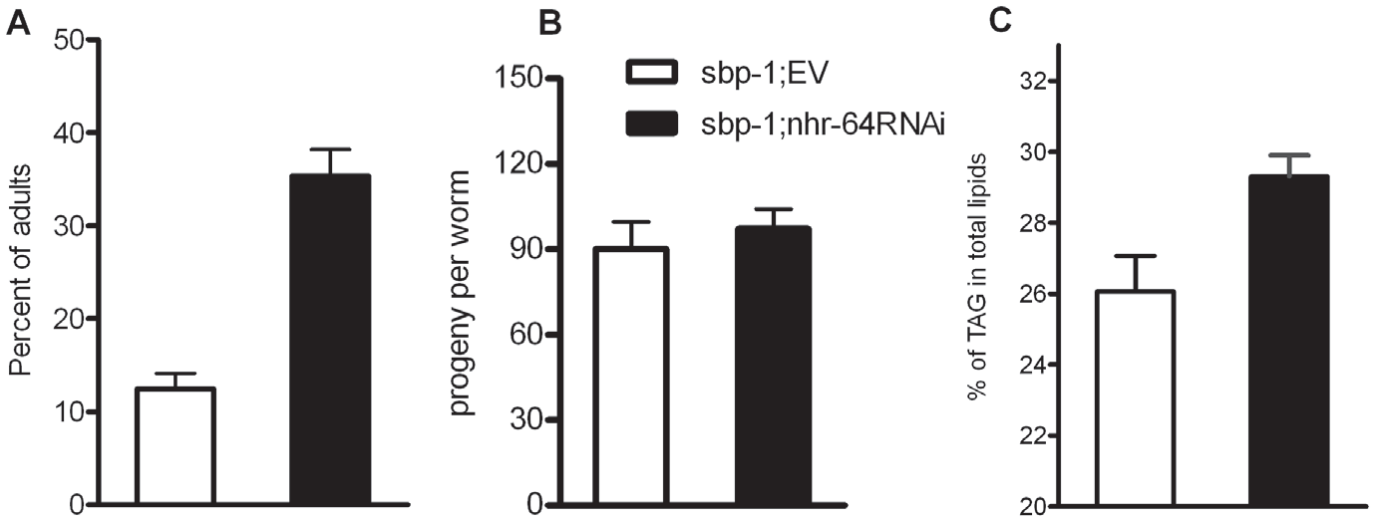
Inactivation of *nhr-64* suppressed the growth rate and fat storage but not brood size of *sbp-1(ep79)* mutant. For all panels, white bars are *sbp-1(ep79)*; empty vector and black bars are *sbp-1(ep79);nhr-64(RNAi)* grown on *E. coli* strain HT115. (A) Inactivation of *nhr-64* improved the growth of *sbp-1(ep79)* animals. The number of animals that reached adult stage were counted 75 h after plating synchronized L1 larvae. 500–600 worms were scored for each genotype. Error bars are SEM. (B) The average number of progeny produced by individual *sbp-1(ep79)* animals treated with either empty vector (EV) or *nhr-64RNAi*, n = 25 individuals for each treatment. Error bars are SEM. (C) Inactivation of *nhr-64* increased the triacylglycerol/total lipid ratio in *sbp-1* mutants. Total lipids were extracted from two independent biological replicates and separated into triacylglycerol and phospholipid fractions using thin layer chromatography and quantified using gas chromatography. Error bars show the range of two independent biological replicates.

A strain carrying a *sbp-1* deletion allele (*ep79*) was created by excision of a Tc1 element, resulting in removal of the C-terminal regulatory regions of the gene that deletes 2181 bp of the gene (Figure S1A). This *sbp-1(ep79)* strain displays a phenotype very similar to *sbp-1RNAi*. The animals grow slowly, and have decreased fat stores and altered fatty acid composition compared to wild type (Figures S1B and S1C), indicating that this deletion is a reduction-of-function allele [7], [17], [18], [24]. We treated *sbp-1(ep79)* mutants with *nhr-64RNAi* and observed a faster growth rate than the empty vector controls (Figure 2A). However, brood size did not increase (Figure 2B). Consistent with previous reports [7], [17], [18], [24], *sbp-1(ep79)* mutant displayed fewer lipid droplets in both intestinal and hypodermal cells compared to wild type, but inactivation of *nhr-64* increased the amount of TAG stores in *sbp-1(ep79)* (Figure 2C). Our data indicate that *nhr-64RNAi* can partially suppress the slow growth rate and low fat stores of *sbp-1(ep79)* mutants.

### *nhr-64*RNAi affects fatty acids composition of monomethyl branched chain fatty acids and stearic acid

**Figure 3:**
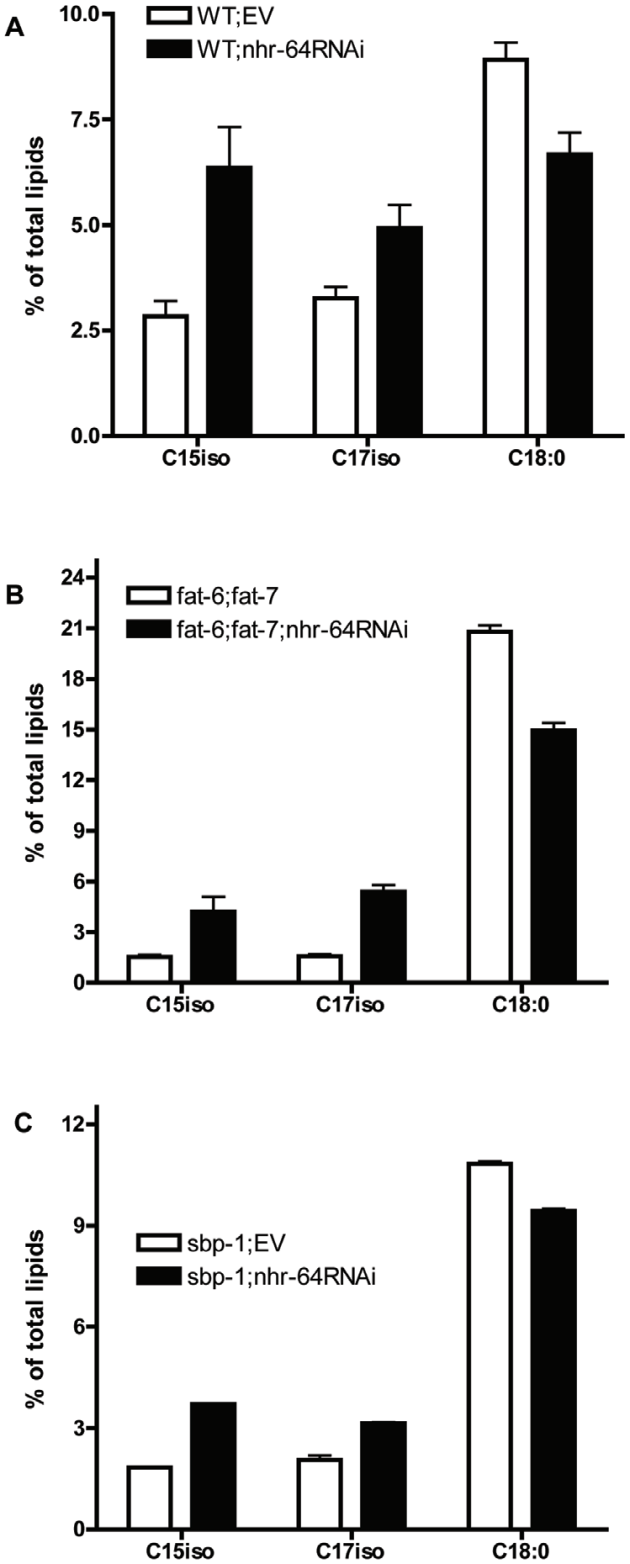
Inactivation of *nhr-64* by RNAi influences fatty acid composition. Treatment with *nhr-64RNAi* led to increased levels of mono-methyl branched fatty acids C15iso and C17iso and reduced levels of C18∶0 in (A) wild type (B) *fat-6;fat-7* double mutants and (C) *sbp-1(ep79)* mutants. Values are obtained from total lipids measured by gas chromatography (GC), worms were grown on *E. coli* strain HT115. Data shown are the average of three or four determinations of total fatty acids, each from two or three independent biological replicates; error bars represent the standard error. All comparisons shown were determined to differ significantly, P<0.05.

Because reduction of *nhr-64* by RNAi increases fat stores in wild type, *fat-6;fat-7* double mutants, and *sbp-1* mutants, we asked whether inactivation of *nhr-64* influences fatty acid composition. We found reproducible changes in fatty acid composition of wild type, *fat-6;fat-7*, and *sbp-1* mutants treated with *nhr-64RNAi* (Figure 3). In all three strains, we observed increases in the levels of two monomethyl branched-fatty acids (mmBFAs), C15iso and C17iso, in *nhr-64RNAi* treated worms, while levels of stearic acid (18∶0) decreased. The relative percentages of other fatty acids, including levels of unusual polyunsaturated fatty acids produced by *fat-6;fat-7* double mutants [23] did not change.

Given the importance of C15iso and C17iso in the regulation of growth and development in *C. elegans* [24], [25], as well as the fact that *fat-6;fat-7* and *sbp-1* mutants have low levels of C15iso and C17iso [23], [24], we suspected that improved growth and higher fat stores of *fat-6;fat-7* double mutants treated with *nhr-64RNAi* might be a consequence of increased levels of C15iso and C17iso. However, dietary supplementation with a range of concentrations of C15iso, C17iso, or a combination of both fatty acids did not improve growth rate or fat stores of *fat-6;fat-7* double mutants, even though the dietary supplementation successfully increased the levels of C15iso and C17iso in the worms (data not shown). Alternatively, optimal levels of stearic acid (18∶0) may be critical for proper fat storage, growth, and reproduction [10]. We found that the decrease in 18∶0 correlates with improved growth and increased fat storage in the *fat-6;fat-7* and *sbp-1* mutants treated with *nhr-64(RNAi)*.

### Two *nhr-64* deletion mutations produce truncated NHR-64 with residual transcriptional activity

To confirm the suppression of *fat-6;fat-7* double mutants by inactivation of *nhr-64*, we examined two available *nhr-64* deletion mutants, *ok1957* and *tm1106*. Both *nhr-64(ok1957)* and *nhr-64(tm1106)* mutants appear similar to wild type, with no obvious changes in growth rate, brood size, and fat storage. The only phenotype shared by the deletion mutants and the *nhr-64RNAi* were specific fatty acid composition changes, most notably the higher levels of C15iso and C17iso fatty acids (Figure S2A). We generated both *nhr-64;fat-6;fat-7* triple mutant strains and found that, growing on *E. coli* OP50, neither deletion mutant was able to suppress the fat storage and growth defects in *fat-6;fat-7* double mutants. In fact, the triple mutant strains were indistinguishable from the *fat-6;fat-7* double mutants with respect to fat stores, growth rate, and brood size. The triple mutants did contain higher C15iso and C17iso levels than *fat-6;fat-7*, but only a slight reduction in 18∶0 (Figure S2B and data not shown). Furthermore, treating the *nhr-64;fat-6;fat-7* strain with RNAi corresponding to the full-length *nhr-64* coding sequence led to improved growth rate, increased brood size, and reduced 18∶0 content.

We considered the possibility that our *nhr-64* RNAi construct might target other nuclear hormone receptors, and that suppression was due to reduction of more than one NHR. However, the predicted sequence of *nhr-64* revealed that the closest homolog, *nhr-69* shows merely a 42% identity, with no stretches greater than 11 nucleotides of identity. Furthermore, we observed no improvement of fat storage, growth rate, and brood size of *fat-6;fat-7* when treated with RNAi corresponding to *nhr-69*.

In order to more directly address the issue of off-target effects of *nhr-64*RNAi, we constructed an additional RNAi feeding construct corresponding to 1,184 base pairs of the *nhr-64* gene. We chose the region of *nhr-64* which is deleted in the *nhr-64(ok1957)* and *nhr-64(tm1106)* mutant strains, so that the feeding RNAi would not be able to deplete any residual *nhr-64* transcript that may be present in the deletion mutants. We found that while the truncated *nhr-64*RNAi construct was able to suppress the slow growth and low brood size of *fat-6;fat-7* to a similar degree as the full-length *nhr-64*RNAi construct, it had no affect on the growth rate or brood size of the *nhr-64;fat-6;fat-7* strain (data not shown). Thus, it is unlikely that off target RNAi is occurring and we sought to test whether the protein produced by the truncated *nhr-64* gene may be partially functional.

**Figure 4:**
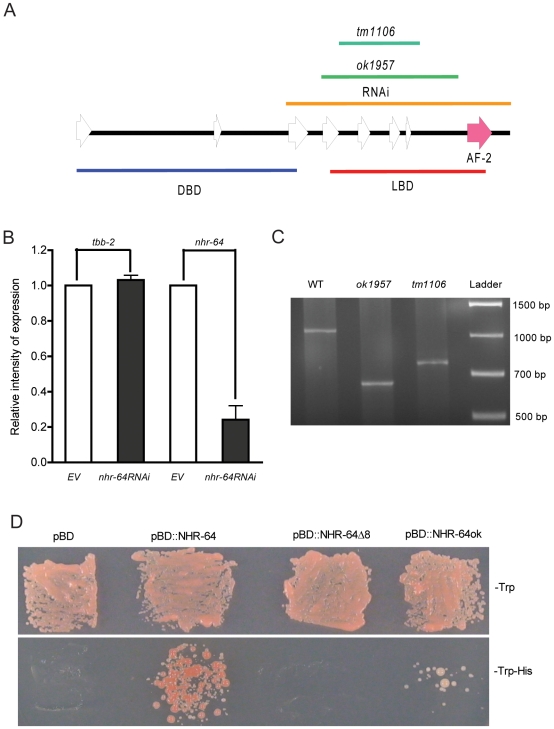
Partial suppression and partial transcriptional activation activity of an *nhr-64* deletion mutant. (A) Simplified scheme of *nhr-64* gene structure. White arrows are exons and black lines are introns. DBD: DNA binding domain; LBD: ligand binding domain. RNAi: RNA interference; AF-2: transactivation domain. (B) The expression of *nhr-64* in L4s WT animals treated with *nhr-64RNAi* or EV control. Data were the average of two independent biological repeats and quantified with GeneSnap software. (C) The transcripts of *nhr-64* in WT (1110 bp), *nhr-64(ok1957)* mutation (665 bp) and *nhr-64(tm1106)* mutation (771 bp). Ladder: 1 kb DNA ladder (Fisher Scientific). (D) Demonstration of transcriptional activation activity of WT and truncated NHR-64. Deletion of exon 8 (pBD::NHR-64Δ8) abolished the ability of NHR-64 to grow on selective SD medium without both tryptophan and histidine (SD-trp-his), indicating lack of transcriptional activation activity. The protein predicted to be synthesized by the *nhr-64(ok1957)* deletion mutant (pBD::NHR-64ok) retains the ability to grow on SD-trp-his selective medium, indicating residual transcriptional activation activity.

The first three exons of *nhr-64* encode a predicted DNA binding domain (DBD) and the remaining 5 exons encode a predicted ligand binding domain (LBD) [26], [27]. While part of the predicted LBD is deleted in both *nhr-64* mutations (Figure 4A), transcripts generated by the deletion mutants are predicted to contain exon 8, which encodes the C-terminal activation domain 2 (AF-2), a region crucial for transcriptional activity [28], [29]. This domain is conserved in *nhr-64*, including amino acids required for efficient transcriptional activation (Figure S3). Furthermore, the AF-2 domain is expected to be translated in the mutant strains because both deletions permitted the reading frame to be unchanged. Indeed, RT-PCR analysis showed not only that the expression of *nhr-64* was significantly reduced by *nhr-6*4 RNAi compared to empty vector background (Figure 4B), but that the mutant *nhr-64* strains express shorter *nhr-64* transcripts at similar levels as wild type (Figure 4C). Sequence analysis revealed that transcripts produced by both *nhr-64* deletion strains contain the DNA binding domain (DBD) and in-frame, intact sequence of exon 8, including an intact AF-2 domain.

To determine if NHR-64 can function as a transcriptional activator, we tested activity in yeast using the GAL4BD expression system. We fused the full-length NHR-64 and truncated NHR-64 lacking the putative transcriptional activation domain, together with yeast GAL4BD, and expressed the fusion proteins in yeast. If NHR-64 has transcriptional activity, it will interact with the GALBD to bind the promoter and activate expression of the HIS reporter gene. This allows the yeast strain carrying the NHR-64 fusion protein to grow on selective media lacking histidine. We found that yeast containing the GALBD::NHR-64 fusion protein grew on media lacking histidine, while yeast containing the GALBD construct alone, as well as yeast carrying the truncated NHR-64 lacking the AF-2 domain, were unable to grow on media without histidine (Figure 4D). Furthermore, the fusion protein corresponding to the *ok1957* deletion of NHR-64 was also able to grow on media lacking histidine, although the yeast did not grow as well as those carrying the wild-type NHR-64 fusion protein (Figure 4D). These results show that NHR-64 possesses transcriptional activating activity, and that this activity depends on the C-terminal transactivation domain. Furthermore, the deleted NHR-64, lacking part of the LBD, retains residual transcription activity. This finding offers an explanation as to why the *nhr-64* deletion mutants do not show the extent of suppression of *fat-6;fat-7* low fat stores or slow growth as treatment with *nhr-64(RNAi)*.

### Potential gene targets of NHR-64

**Figure 5:**
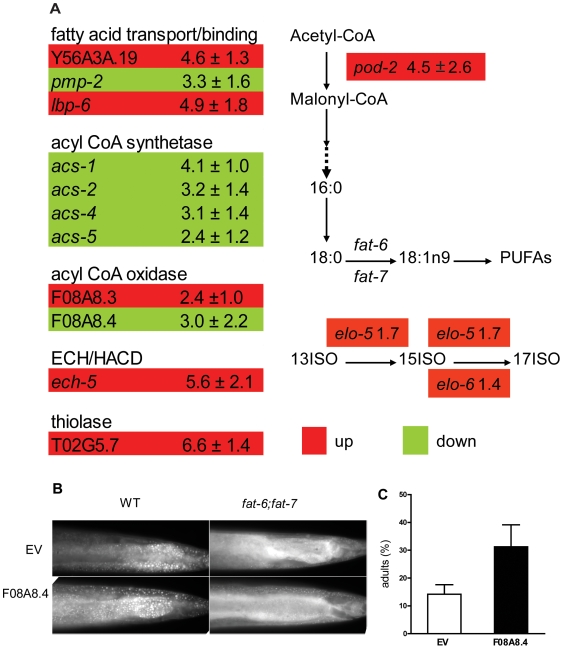
NHR-64 affects transcription of metabolic genes. (A) Quantitative RT-PCR was use to investigate the expression of 89 metabolic genes in L4 stage nematodes treated with *nhr-64RNAi* compared to empty vector controls. Worms were grown on *E. coli* strain HT115. For full list of genes tested, see Table S1. Data shown are the average of three or four biological determinations. (B) Fat staining of fixed wild-type and *fat-6;fat-7* young adults treated with RNAi corresponding to F08A8.4 shows that depletion of the acyl-CoA oxidase encoded by F08A8.4 leads to increased fat stores. Anterior is left and posterior is right. (C) Inactivation of F08A8.4 in *fat-6;fat-7* double mutants resulted in increased growth rate. The number of worms that had reached adult stage in a population were counted 72 hours after plating synchronized L1 stage larvae. The experiment was repeated three times, each time with 100–200 worms. Error bars are SEM.

Because NHR-64 displays transcriptional activation activity, we explored potential target genes by using real time quantitative RT-PCR to measure the expression of 89 genes predicted to be involved in fat metabolism. L4 stage wild-type worms were fed bacteria expressing double stranded RNA corresponding to *nhr-64* or empty vector controls [10], [23]. We found reproducible changes in 14 of the genes, with six showing decreased expression and eight showing increased expression in *nhr-64RNAi* worms compared to empty vector controls (Figure 5A and Table S1). A major gene expression change consistent with higher *de novo* fat synthesis in *nhr-64* worms is a 4.5 fold increase in expression of *pod-2* (W09B6.1), which encodes acetyl-CoA carboxylase, the rate limiting step of fatty acid biosynthesis [30]. Increased expression of acetyl-CoA carboxylase, together with subtle increases in expression of *elo-5* (1.7 fold) and *elo-6* (1.4 fold), are consistent with higher levels of C15iso and C17iso observed in the *nhr-64RNAi* animals. Although the yeast studies provide evidence that NHR-64 is a transcriptional activator, nuclear receptors may also act as repressors, depending on their interactions with ligands, corepressors, or binding partners [26], [27].

One potential target of NHR-64 activation is F08A8.4, which encodes a protein homologous to acyl CoA oxidase, an enzyme that participates in oxidation of fatty acids in peroxisomes [31]. F08A8.4 is expressed three fold lower in *nhr-64RNAi* worms than in WT worms. Furthermore, when it is depleted by RNAi, fat droplets in wild type and in wild type and *fat-6;fat-7* increase (Figure 5B). Importantly, RNAi of F08A8.4 also improved the growth rate of *fat-6;fat-7* double mutants (Figure 5C). Another gene with reduced expression in *nhr-64RNAi* is also predicted to act in peroxisomes. The *pmp-2* gene encodes a protein similar to ABC transporters predicted to transport long-chain fatty acids into peroxisomes.

We then fed worms *E. coli* expressing double stranded RNA corresponding to the genes encoding the other six acyl-CoA oxidase isoforms, as well as on the two peroxisomal bifunctional enzymes *ech-8* and *ech-9*, and finally on *dhs-28*, a recently characterized component of peroxisomal fatty acid oxidation that is required for the biosynthesize of daumone, the dauer-inducing pheromone. There was no improvement of *fat-6;fat-7* growth rate or fat stores when these mutants were treated with RNAi corresponding to the additional acyl-CoA oxidases, either of the bifunctional enzymes, or *dhs-28*. These studies indicate that F08A8.4 is a key, regulated enzyme in the peroxisomal β-oxidation pathway.

Four acyl-CoA synthetase genes, *acs-1*, *acs-2*, *acs-4*, and *acs-5* also show decreased expression in *nhr-64 RNAi* treated worms. Acyl-CoA synthetases activate fatty acids for β-oxidation, but also for synthetic processes such as fatty acid desaturation, fatty acid elongation, phospholipid synthesis and TAG synthesis [32]. It is thought that various isoforms expressed in particular organelles and tissues play specific roles in channeling fatty acids into degradative or synthetic pathways. Previous studies have shown that the expression of *acs-2* is activated by NHR-49 in mitochondria, where its activity promotes β-oxidation[10]. Similarly, inactivation of *acs-4* represses serotonin-induced fat reduction, indicating that ACS-4 also activates fatty acids for β-oxidation [33].

Finally, several genes predicted to encode components of β-oxidation machinery, including an enoyl-CoA hydratases, an acyl CoA oxidase, and a thiolase showed increased expression in *nhr-64RNAi* nematodes compared to controls. This gene expression pattern seems contradictory to the high fat stores in *nhr-64RNAi*, however, given the complexity and redundancy of the fat-regulatory system, it is plausible that these genes are activated indirectly to compensate for metabolic changes induced by the depletion of NHR-64.

## Discussion

Energy homeostasis depends on proper control of the balance between fat synthesis and fat oxidation. Stearoyl-CoA desaturase deficient worms (*fat-6;fat-7* double mutants), as well as SREBP deficient worms (*sbp-1* mutants) have lower fat stores, slower growth, and reduced fertility compared to wild type. In addition, the *fat-6;fat-7* strain displays increased expression of multiple genes encoding proteins that are predicted to function in fat oxidation pathways [23]. Depleting *nhr-64* by RNAi leads to higher fat stores and improved growth and reproduction in *fat-6;fat-7* and *sbp-1* mutants, as well as to subtle changes in fatty acid composition and fat storage in wild-type animals (Figures 1–3). Because depletion of *nhr-64* leads to increased expression *pod-2* (acetyl-CoA carboxylase), the rate limiting step of fatty acid synthesis, and also to higher levels of the momonmethyl branched chain fatty acids C15iso and C17iso (Figure 5A), one function of NHR-64 may be to act as a repressor of fat synthesis. Indeed, higher levels of C15iso and C17iso may be indicative of higher levels of endogenous fat synthesis [34]. At the same time, depletion of *nhr-64* causes increased expression of acyl-CoA oxidase, which functions in peroxisomal fat oxidation. Depletion of this acyl-CoA oxidase itself causes higher fat stores in wild type and *fat-6;fat-7* animals, and improves the growth rate of *fat-6;fat-7* (Figure 5). Experiments in yeast support the assertion that NHR-64 can function as a transcriptional activator (Figure 4D).

One model to explain the suppression of *fat-6;fat-7* by *nhr-64* is that the two are acting in parallel pathways, with each pathway having an opposite effect on the fat storage/fat oxidation balance. Because reducing stearoyl-CoA desaturase activity drives the balance toward oxidation, and reducing NHR-64 activity drives the balance back toward storage, a more optimal outcome will occur when activity of both pathways is reduced. An alternative model is that 18∶0 (which accumulates to high levels in *fat-6;fat-7* mutants), or a signal derived from it, may activate NHR-64 to promote increased fat oxidation in peroxisomes. Indeed, mammalian nuclear receptors such as PPAR-α and PPAR-γ are activated by fatty acids or their derivatives [26], [27] and structural analysis of the ligand binding domain of HNF4α revealed long chain fatty acids bound in the ligand binding pocket [35], [36].

Even though the *nhr-64* deletion mutants contain elevated levels of monomethyl branch chain fatty acids, which may suggest increased fat synthesis, the mutants failed to suppress the low fat stores or slow growth of *fat-6;fat-7* double mutants. The fact that the *nhr-64* deletion strains fail to increase the fat stores in the *fat-6;fat-7* double mutants growing on these strains indicates that the truncated NHR-64 protein may retain the ability to activate transcription of the peroxisomal fat oxidation genes. Therefore, decreased fat oxidation in *nhr-64* depleted *fat-6;fat-7* mutants may be the key process necessary for the suppression of the low fat stores and slow growth in the stearoyl-CoA desaturase-deficient strain.

Notably, depletion of NHR-64 by RNAi not only increased fat stores in *fat-6;fat-7* and *sbp-1* mutants, but also permitted a faster growth rate in both strains. One contributor to the low fat phenotype of stearoyl-CoA deficiency in mice and nematodes is likely to be increased fat oxidation [21], [23]. Our studies suggest that fatty acid oxidation is reduced in the absence of NHR-64, and therefore the fats that avoid oxidation may be available as an energy source for organismal growth. Alternatively, the altered fatty acid composition, especially reduced 18∶0 in *nhr-64(RNAi);fat-6;fat-7* compared to *fat-6;fat-7* may allow for faster growth because of a more optimal membrane lipid composition. Both *fat-6;fat-7* and *sbp-1* strains have greatly reduced fecundity. It is interesting that *nhr-64* depletion led to the the production of more viable embryos in the *fat-6;fat-7* strain, but it did not improve fecundity of the *sbp-1* strain. This suggests that increased fat stores *per se* is not sufficient to overcome the reproductive defects in the *sbp-1* strain.

NHR-64 is the third member of the expanded family of *C. elegans* HNF4 nuclear receptors shown to regulate lipid metabolism. NHR-49 and NHR-80 are both necessary for efficient transcription of stearoyl-CoA desaturase genes and are therefore required for maintaining proper fatty acid composition [10], [11]. NHR-64 is one of five *C. elegans* NHRs that shows relatively strong similarity to the NHR-49 ligand binding domain (LBD), and, of these five, only NHR-64-LBD was shown to interact with the transcriptional mediator subunit MDT-15 in a yeast two-hybrid assay [37]. MDT-15 also interacts with SREBP, and the fatty acid composition analysis *C. elegans* depleted of MDT-15 by RNAi reveals a severe deficiency in unsaturated fatty acids [18], [37]. Combining *fat-6* mutants with *nhr-49* or *sbp-1* leads to lethality due to the lack of compensatory up-regulation of *fat-5* and *fat-7* genes (our unpublished observations), however, this study reveals that depletion of *nhr-64* leads to suppression of the *fat-6;fat-7* low fat and slow growth phenotypes, and that the improved growth rate and lower fat stores correlates with lower stearic acid content. Thus, various members of the *C. elegans* HNF4 nuclear receptor family are capable of responding in opposite ways to maintain optimal lipid homeostasis. Opposing activities of nuclear receptors are critical for ensuring lipid homeostasis in mammals, for example, PPARα promotes β-oxidation of fatty acids, while PPARγ promotes adipocyte differentiation and fat storage [38].

We have identified NHR-64 as a novel regulator of fat homeostasis in *C. elegans*. These studies underscore the complexity of compensatory mechanisms that occur in animals to balance fat storage, growth, and reproductive efficiency. We propose that NHR-64 inhibits *de novo* fat synthesis, and under certain conditions, acts to promote fat oxidation in peroxisomes. Proper function of NHR-64 maintains optimal fat stores for growth and reproduction.

## Materials and Methods

### Worm strains, growth conditions and RNAi

Nematode growth media was used to maintain *C. elegans* with the *E. coli* (OP50) as food at 20° unless specifically noted. The wild-type strain was N2. The strains used in this study were: RB1592 *nhr-64(ok1957)*, BX211 *nhr-64(tm1106)*, CE541 *sbp-1(ep79)*, BX156 *fat-6(tm331);fat-7(wa36)*, BX212 *nhr-64(tm1106);fat-6(tm331);fat-7(wa36)*, *BX202 nhr-64(ok1957);fat-6(tm331);fat-7(wa36)*. Dietary fatty acid supplementation experiments used freshly prepared plates as described in [39]. RNAi was performed by feeding bacterial strains from the Ahringer *C. elegans* RNAi library, obtained from Gene Services (Source Bioscience) [40]. The empty vector (L4440) in the Ahringer library HT115 *E. coli* strain was used as the negative control for RNAi experiments. The *nhr-64* deletion RNAi construct was made by amplifying 1,184 base pairs of *C. elegans* genomic DNA corresponding to the region of *nhr-64* that is deleted in the *nhr-64(ok1957)* and *nhr-64(tm2206)* strains, using the forward primer (CTCGTAAACAGGCGACCACA) and the reverse primer (AATCGGTAAGCCGTTCA). The amplified sequence was cloned into the “double T7” plasmid pPD129.36 [41] and transformed into *E. coli* HT115.

### Fatty acid composition and lipids analysis

Fatty acid composition of adult nematodes was determined as previously described [11], [23], [42]. For determination of triacylglycerol and phospholipids, lipid extraction and thin-layer chromatography was performed as described in [7] and [39].

### Nile Red staining of fixed nematodes

L4s or young adults nematodes were washed off of growth plates, fixed and stained with Nile Red as described in [43]. Images were captured using identical settings and exposure time for each image.

### Growth rate analysis

Eggs were isolated from gravid adults using hypochlorite treatment and hatched in M9 buffer overnight, and then plated onto NGM plates seeded with *E. coli* strain HT115 carrying empty vector or *nhr-64RNAi*. The number of worms that reached adult stage was scored 3 days later.

### Fertility analysis

L1 larvae were plated onto RNAi plates seeded with *E. coli* strain HT115 carrying empty vector or *nhr-64RNAi* and allowed to develop to L4 stage. At this time 10–15 L4 worms were transferred individually to fresh RNAi plates. Worms were transferred daily until they did not produce any more progeny. Two or three days after removal of the adult, the number of live progeny was counted.

### Quantitative RT–PCR analysis

The Quantitative RT-PCR protocol was modified from *Brock et al.* [23]. Generally, L4s nematodes grown on *E. coli* HT115 were harvested and RNA was prepared using TRIzol Reagent (Invitrogen, San Diego). A DNA-FREE RNA kit (Zymo Research) was used for DNase treatment and purification. After quantification, 1 µg of total RNA was used in a reverse-transcription reaction with SuperScript III (Invitrogen) to generate cDNA. Primer sequences for the metabolism genes were obtained from Marc Van Gilst [10]. The PCR mixture consisted of 0.3 µm primers, cDNA, ROX, and 1× SYBR green mix (Invitrogen Platinum SYBR green qPCR Supermix UDG). The quantitative RT–PCR (QRT–PCR) was run and monitored on a MX3000P machine (Stratagene, La Jolla, CA). Relative abundance was determined using the ΔΔCt method and the reference genes *tbb-2* and *ubc-2* to control for template levels.

### RT-PCR of *nhr-64*

Total RNA was prepared and cDNA was generated as previously described. The following primers were used to amplify the full-length *nhr-64* cDNA. Forward primer: 5′-CACCATGACACTGGAAGAAAAAG-3′; reverse primer:5′-TTATTGATGGCACATAATTGG-3′. Polymerase chain reaction (PCR) was carried out using the *TaKaRa Ex Taq* system (Takara Bio Inc, Japan), which included 10 mM (2.5 mM each) (dNTPs), 10× *Ex Taq* Buffer (20 mM Mg^2+^ plus), and 5 U/µL *TaKaRa Ex Taq* polymerase. Each 25 microliter PCR reaction mix contained 1× *Ex Taq* buffer, 1 mM total concentration of *TaKaRa* dNTP mixture, 0.5 U *TaKaRa Ex Taq*, and 1.0 µM of each primer, 100 ng cDNA as template. PCR conditions were 1 cycle of 3 min at 95°C, followed by 22 cycles of 40 sec at 95°C, 20 sec at 50°C, and 2 min at 72°C, and finishing with 10 min incubation at 72°C.

### Transcription activity of NHR-64

Wild-type and truncated NHR-64 without exon 8 were amplified by PCR using *TaKaRa Ex Taq* system (Takara Bio Inc, Japan) and mixed-stage mixed-stage WILD-TYPE *C. elegans* cDNA as template. Truncated NHR-64(ok) was amplified from mixed-stage *nhr-64(ok1957)* mutant cDNA as template (primer sequences are available upon request). The resulting cDNAs were sequenced and subcloned into pENTR/D-TOPO® vector (Invitrogen), and then transferred to modified pBD-GAL4 Cam Vector (Stratagene) by RL recombination reaction, to generate plasmids expressing individual GAL4-DBD-NHR-64, GAL4-DBD-NHR-64Δ8 and GAL4-DBD-NHR-64(ok) fusions. Plasmids were transformed into yeast strain YRG-2, which contains LacZ and HIS3 reporter genes, using standard PEG3350/LiAc methods. SD medium without tryptophan and histidine were used to select the transcriptional activity.

## Supporting Information

Figure S1 Characterization of *sbp-1(ep79)*. (A) Simplified scheme of *sbp-1* gene structure. White arrows are exons and black lines are introns. *sbp-1* consists of 13 exons. Exon 6 of *sbp-1* encodes a helix-loop-helix (HLH) domain indicated by red rectangle and *ep79* deletion removed 2181 base pairs indicated by blue rectangle. (B) Fatty acid composition of wild type and *sbp-1(ep79)*. Only fatty acids showing significant differences with wild type are shown. MMBC  =  monomethyl branch chain fatty acids (sum of C15iso and C17iso). (C) Relative amount of triacylglycerol/total lipid in *sbp-1(ep79)* compared to wild type. Total lipids were extracted from nematodes grown on *E. coli* strain OP50 and separated into triacylglycerol and phospholipid fractions using thin layer chromatography, fractions were quantified using gas chromatography.
(1.21 MB TIF)

Figure S2 Fatty acid composition of an *nhr-64* mutant grown on *E. coli* strain OP50. (A) The *nhr-64(ok1957)* mutant strain contains increased levels of C15iso and C17iso and decreased C18:0 compared to wild type. (B) The *fat-6;fat-7;nhr-64(ok1957)* triple mutant strain contains increased levels of C15iso and C17iso and decreased C18:0 compared to the *fat-6;fat-7* double mutant strain. Values shown are mean and SEM of four determinations. The difference in mean amount of all fatty acids shown were found to be statistically significant by student's T test (P<0.05).
(0.87 MB TIF)

Figure S3 Amino acid alignment of HNF4 genes. Shown are human HsHF4 (NP_849180), mouse MmHF4(NP_032287), Drosophila DeHF4 (NP_476887), and *C. elegans* NHR-64(AAC24283). DBD: DNA binding domain; LBD: ligand binding domain. E: Glutamic acid residue; L: Leucine residue. Sequences of amino acid covered with gray color are encoded by exon 8. The glutamic acid residue (E) and leucine residue (L) marked by red are highly conserved in human, mouse, and Drosophila and *C. elegans*.
(1.41 MB TIF)

Table S1 Genes analyzed in quantitative RT-PCR experiments.
(0.11 MB DOC)
