# Supplementary figures and images for: The Role of Nuclear Receptor NHR-64 in Fat Storage Regulation in Caenorhabditis elegans
Source: PLoS One. 2010 Mar 25;5(3):e9869. doi: 10.1371/journal.pone.0009869 (PMC2845610; doi:10.1371/journal.pone.0009869)

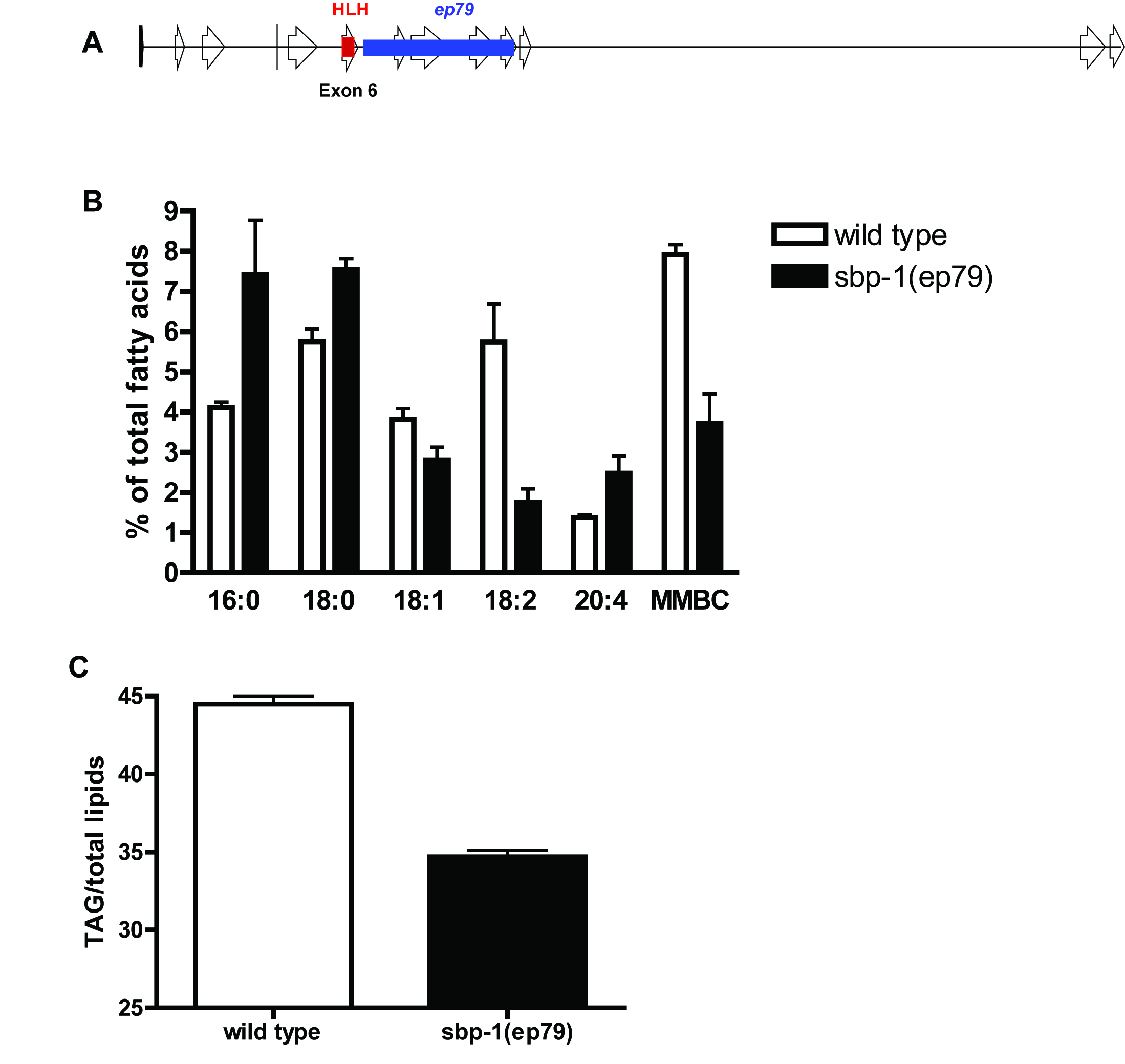

Supplement: Figure S1 — Characterization of sbp-1(ep79). (A) Simplified scheme of sbp-1 gene structure. White arrows are exons and black lines are introns. sbp-1 consists of 13 exons. Exon 6 of sbp-1 encodes a helix-loop-helix (HLH) domain indicated by red rectangle and ep79 deletion removed 2181 base pairs indicated by blue rectangle. (B) Fatty acid composition of wild type and sbp-1(ep79). Only fatty acids showing significant differences with wild type are shown. MMBC = monomethyl branch chain fatty acids (sum of C15iso and C17iso). (C) Relative amount of triacylglycerol/total lipid in sbp-1(ep79) compared to wild type. Total lipids were extracted from nematodes grown on E. coli strain OP50 and separated into triacylglycerol and phospholipid fractions using thin layer chromatography, fractions were quantified using gas chromatography. (1.21 MB TIF) [file pone.0009869.s001.tif]

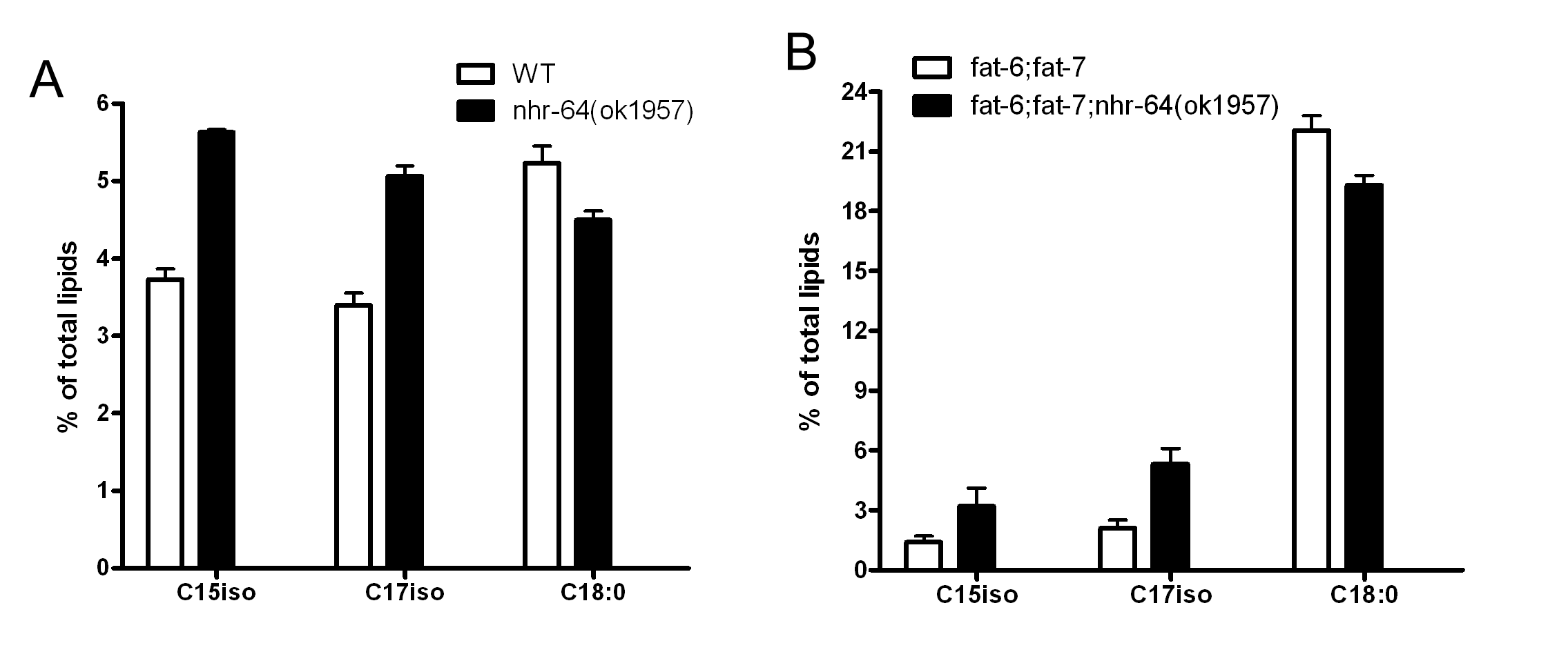

Supplement: Figure S2 — Fatty acid composition of an nhr-64 mutant grown on E. coli strain OP50. (A) The nhr-64(ok1957) mutant strain contains increased levels of C15iso and C17iso and decreased C18:0 compared to wild type. (B) The fat-6;fat-7;nhr-64(ok1957) triple mutant strain contains increased levels of C15iso and C17iso and decreased C18:0 compared to the fat-6;fat-7 double mutant strain. Values shown are mean and SEM of four determinations. The difference in mean amount of all fatty acids shown were found to be statistically significant by student's T test (P<0.05). (0.87 MB TIF) [file pone.0009869.s002.tif]

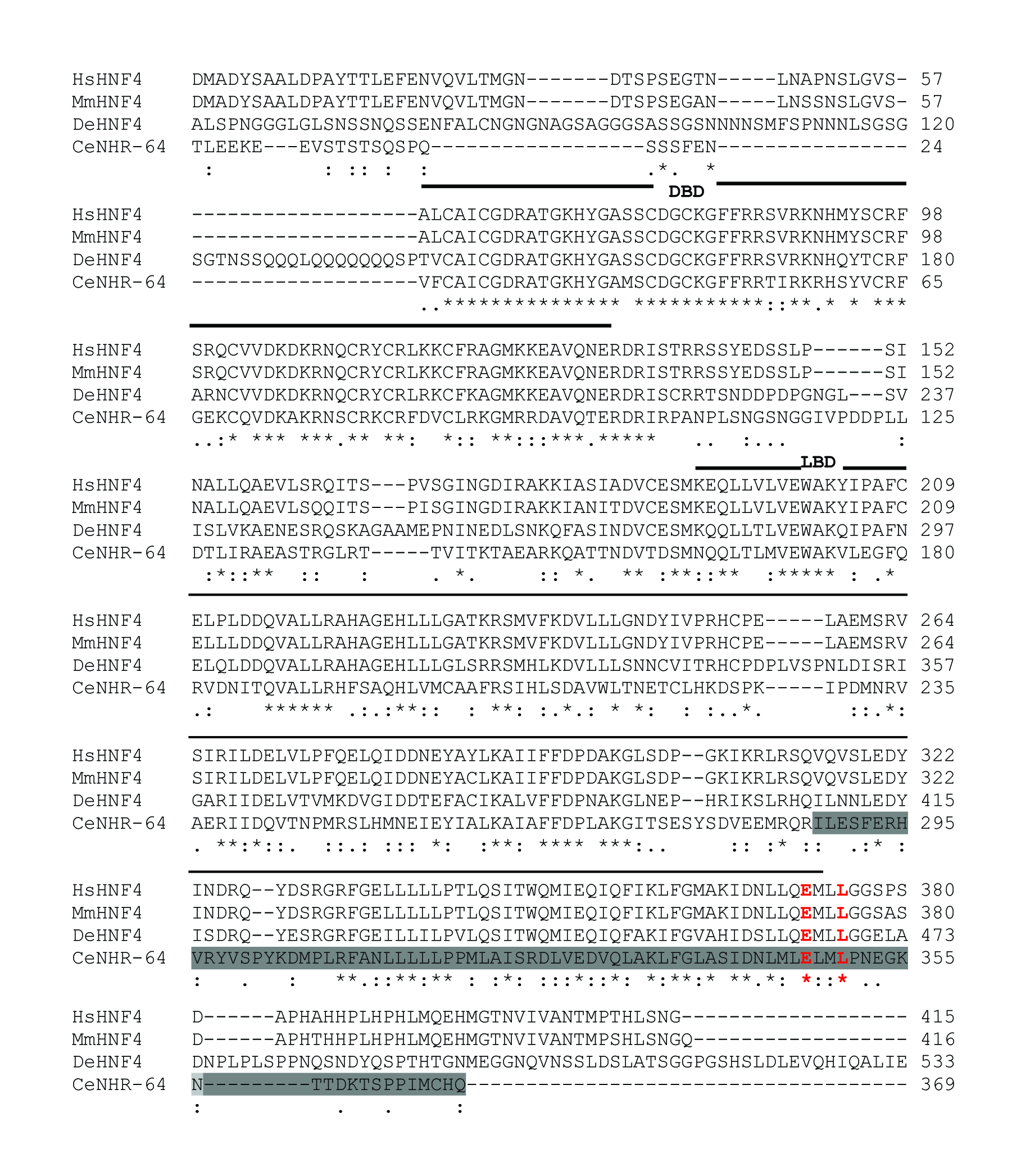

Supplement: Figure S3 — Amino acid alignment of HNF4 genes. Shown are human HsHF4 (NP_849180), mouse MmHF4(NP_032287), Drosophila DeHF4 (NP_476887), and C. elegans NHR-64(AAC24283). DBD: DNA binding domain; LBD: ligand binding domain. E: Glutamic acid residue; L: Leucine residue. Sequences of amino acid covered with gray color are encoded by exon 8. The glutamic acid residue (E) and leucine residue (L) marked by red are highly conserved in human, mouse, and Drosophila and C. elegans. (1.41 MB TIF) [file pone.0009869.s003.tif]
