# Supplementary material for: The Role of Nuclear Receptor NHR-64 in Fat Storage Regulation in Caenorhabditis elegans
Source: PLoS One. 2010 Mar 25;5(3):e9869. doi: 10.1371/journal.pone.0009869 (PMC2845610; doi:10.1371/journal.pone.0009869)
Supplement: Table S1 — Genes analyzed in quantitative RT-PCR experiments. (0.11 MB DOC) [file pone.0009869.s004.doc]

| **Gene** | **GeneID** | **Predicted activity** | **fold change** |
| --- | --- | --- | --- |
| acdh-1 | C55B7.4A | Acyl CoA dehydrogenase | NC |
| acdh-2 | C17C3.12A | Acyl CoA dehydrogenase | NC |
| acdh-3 | K06A5.6 | Acyl CoA dehydrogenase | NC |
| acdh-7 | T25G12.5 | Acyl CoA dehydrogenase | NC |
| acdh-8 | K05F1.3 | Acyl CoA dehydrogenase | **NR** |
| acdh-12 | E04F6.5 | Acyl CoA dehydrogenase | NC |
| ech-1 | C29F3.1 | Enoyl-CoA Hydratase | NC |
| ech-2 | F38H4.8 | Enoyl-CoA Hydratase | **NR** |
| ech-3 | F43H9.1 | Enoyl-CoA Hydratase | **NR** |
| ech-4 | R06F6.9 | Enoyl-CoA Hydratase | NC |
| ech-5 | F56B3.5 | Enoyl-CoA Hydratase | 5.6 |
| ech-6 | T05G5.6 | Enoyl-CoA Hydratase | **NR** |
| ech-7 | Y105E8A.4 | Enoyl-CoA Hydratase | NC |
| ech-8 | F01G10.2 | Enoyl-CoA Hydratase | NC |
| ech-9 | F01G10.3 | Enoyl-CoA Hydratase | NC |
|  | B0272.3 | 3OH-Acyl CoA Dehydrogenase | NC |
| ard-1 | F01G4.2 | 3OH-Acyl CoA Dehydrogenase | NC |
|  | F54C8.1 | 3OH-Acyl CoA Dehydrogenase | NC |
| hacd-1 | R09B5.6 | 3OH-Acyl CoA Dehydrogenase | **NR** |
|  | T02G5.7 | KetoAcyl-CoA Thiolase | 6.6 |
| kat-1 | T02G5.8 | KetoAcyl-CoA Thiolase | NC |
|  | T02G5.4 | KetoAcyl-CoA Thiolase | NC |
|  | F53A2.7 | KetoAcyl-CoA Thiolase | NC |
|  | B0303.3 | KetoAcyl-CoA Thiolase | NC |
|  | F08A8.3 | Acyl-CoA Oxidase | 2.4 |
|  | F08A8.4 | Acyl-CoA Oxidase | -3.0 |
|  | C48B4.1 | Acyl-CoA Oxidase-1 | NC |
|  | F08A8.1 | Acyl-CoA Oxidase-1 | **NR** |
|  | F08A8.2 | Acyl-CoA Oxidase-1 | NC |
|  | F25C8.1 | Acyl-CoA Oxidase-1 | NC |
|  | F59F4.1 | Acyl-CoA Oxidase-1 | NC |
| ctl-1 | ctl-1 | Catalase | NC |
| ctl-2 | ctl-2 | Catalase | NC |
| ctl-3 | ctl-3 | Catalase | NC |
| pmp-1 | C44B7.8 | ABC transporter | **NR** |
| pmp-2 | C44B7.9 | ABC transporter | -3.3 |
| cpt-1 | Y46G5A.17 | Carnitine Palmitoyl Transferase I | **NR** |
| cpt-2 | R07H5.2 | Carnitine Palmitoyl Transferase II | NC |
| cpt-3 | Y48G9A.10 | Carnitine palmitoyl transferase | NC |
| ctp-4 | K11D12.4 | Carnitine palmitoyl transferase | NC |
|  | T20B3.1 | Carnitine palmitoyl transferase | NC |
|  | C03H5.4 | Phospholipase A2 | NC |
|  | C07E3.9 | Phospholipase A2 | NC |
|  | C46C11.1 | Hormone Sensitive Lipase | NC |
|  |  |  |  |
| acs-1 | F46E10.1 | Acyl-CoA Synthetase | -4.1 |
| acs-2 | F28F8.2 | Acyl-CoA Synthetase | -3.2 |
| acs-4 | F37C12.7 | Acyl-CoA Synthetase | -3.1 |
| acs-5 | Y76A2B.3 | Acyl-CoA Synthetase | -2.4 |
| acs-13 | Y65B4BL.5 | Acyl-CoA Synthetase | NC |
| acs-15 | R07C3.4 | Acyl-CoA Synthetase | NC |
| acs-16 | F47G6.2 | Acyl-CoA Synthetase | NC |
| acs-18 | R09E10.3 | Acyl-CoA Synthetase | NC |
| acs-20 | F28D1.9 | Acyl-CoA Synthetase | NC |
|  | R09E10.4 | Acyl-CoA Synthetase | NC |
|  | D1009.1 | Acyl-CoA Synthetase | NC |
|  |  |  |  |
| elo-2 | F11E6.5 | Fatty acid elongase | NC |
| elo-5 | F41H10.7 | Fatty acid elongase | 1.7 |
| elo-6 | F41H10.8 | Fatty acid elongase | 1.4 |
| elo-9 | Y53F4B.2 | Fatty acid elongase | NC |
| fat-1 | Y67H2A.8 | Omega-3 Desaturase | NC |
| fat-2 | W02A2.1 | Delta-12 Desaturase | **NR** |
| fat-3 | W08D2.4 | Delta-6 Desaturase | **NR** |
| fat-4 | T13F2.1 | Delta-5 Desaturase | **NR** |
| fat-5 | W06D12.3 | Delta-9 desaturase | NC |
| fat-6 | VZK822L.1 | Delta-9 desaturase | NC |
| fat-7 | F10D2.9 | Delta-9 desaturase | NC |
|  | F33D4.4 | Sphingolipid desaturase | NC |
|  | C25A1.5 | Sphingolipid Fatty Acid Hydroxylase | NC |
|  |  |  |  |
|  | EEED8.2 | Fatty Acid Binding Protein | NC |
|  | EEED8.3 | Fatty Acid Binding Protein | NC |
| lbp-1 | F40F4.3 | Fatty Acid Binding Protein | NC |
| lbp-2 | F40F4.2 | Fatty Acid Binding Protein | **NR** |
| lbp-3 | F40F4.4 | Fatty Acid Binding Protein | NC |
| lbp-5 | W02D3.7 | Fatty Acid Binding Protein | NC |
| lbp-6 | W02D3.5 | Fatty Acid Binding Protein | 4.9 |
| lbp-7 | T22G5.2 | Fatty Acid Binding Protein | **NR** |
| lbp-8 | T22G5.6 | Fatty Acid Binding Protein | NC |
| lbp-9 | Y40B10A.1 | Fatty Acid Binding Protein | NC |
|  |  |  |  |
|  | T28F3.5 | Acetyl-CoA Carboxylase | NC |
| pod-2 | W09B6.1 | Acetyl-CoA Carboxylase | 4.5 |
|  | Y56A3A.19 | Acyl Carrier Protein | 4.6 |
|  | F08F8.2 | HMG CoA Reductase | NC |
| dhs-25 | F09E10.3 | Short-chain Dehydrogenase | NC |
| let-767 | C56G2.6 | Short-chain Dehydrogenase | NC |
| fasn-1 | F32H2.5 | Fatty acid synthase | NC |
| mboa-2 | H19N07.4 | DGAT1 | NC |
|  | K07B1.4 | MGAT1 | NC |
|  | W01A11.2 | DGAT2/MGAT2 | NC |
|  | F59A1.10 | DGAT2 | NC |
